# Reduced Oblique Effect in Children with Autism Spectrum Disorders (ASD)

**DOI:** 10.3389/fnins.2015.00512

**Published:** 2016-01-21

**Authors:** Olga V. Sysoeva, Maria A. Davletshina, Elena V. Orekhova, Ilia A. Galuta, Tatiana A. Stroganova

**Affiliations:** Autism Research Laboratory, Center for Neurocognitive Research (MEG Center), Moscow State University of Psychology and EducationMoscow, Russia

**Keywords:** visual orientation discrimination, oblique effect, autism spectrum disorders (ASD), children, plasticity, critical period

## Abstract

People are very precise in the discrimination of a line orientation relative to the cardinal (vertical and horizontal) axes, while their orientation discrimination sensitivity along the oblique axes is less refined. This difference in discrimination sensitivity along cardinal and oblique axes is called the “oblique effect.” Given that the oblique effect is a basic feature of visual processing with an early developmental origin, its investigation in children with Autism Spectrum Disorder (ASD) may shed light on the nature of visual sensory abnormalities frequently reported in this population. We examined line orientation sensitivity along oblique and vertical axes in a sample of 26 boys with ASD (IQ > 68) and 38 typically developing (TD) boys aged 7–15 years, as well as in a subsample of carefully IQ-matched ASD and TD participants. Children were asked to detect the direction of tilt of a high-contrast black-and-white grating relative to vertical (90°) or oblique (45°) templates. The oblique effect was reduced in children with ASD as compared to TD participants, irrespective of their IQ. This reduction was due to poor orientation sensitivity along the vertical axis in ASD children, while their ability to discriminate line orientation along the oblique axis was unaffected. We speculate that this deficit in sensitivity to vertical orientation may reflect disrupted mechanisms of early experience-dependent learning that takes place during the critical period for orientation selectivity.

## Introduction

Autism spectrum disorder (ASD) is a disorder characterized by early emerging social and communication impairments, as well as rigid and repetitive patterns of behaviors and interests. Sensory abnormalities have been reported in the original descriptions of autism (Kanner, 1968), and now are among the core ASD symptoms (American Psychiatric Association, 2013). Atypical sensory processing has been observed in ASD within multiple sensory modalities including vision (Davis et al., 2006; Simmons et al., 2009; Marco et al., 2011; Ausderau et al., 2014). Recent studies have shown that even basic visual processes are affected in ASD (Kéïta et al., 2010, 2011; Koh et al., 2010; Greenaway et al., 2013; Latham et al., 2013; Weinger et al., 2014; Jachim et al., 2015; for a review, see Simmons et al., 2009). Since the low-level visual functions are unlikely to be strongly influenced by higher-order variables, e.g., social experience, those functions might more directly reflect the core neural deficits of ASD observed at the cellular or network levels.

Both enhanced, impaired, and normal visual processing in ASD have been reported. Some elementary visual functions, such as Vernier acuity (Latham et al., 2013), temporal resolution (i.e., detection of asynchrony in the onset of two visual signals; Falter et al., 2012), or contrast sensitivity to static low-level visual stimuli (e.g., ability to identify the low-contrast luminance-defined gratings; Bertone et al., 2005; Kéïta et al., 2014) were found to be superior in adults with ASD. On the other hand, performance of children with ASD in the contrast sensitivity task did not differ from normal (Rivest et al., 2013). Another study reported a deteriorated ability to detect a vertical bar over Gaussian noise in children with ASD (Sanchez-Marin and Padilla-Medina, 2008), although intellectual disability of the participants might account for their poor performance in this task. Collinear facilitation—improved detection of central visual stimulus in the presence of collinearly oriented flankers—was found to be either atypically increased (Kéïta et al., 2011) or reduced (Jachim et al., 2015) in adults and adolescents with ASD. Thus, the presence of a particular perceptual advantage and/or difficulty in ASD seem to depend upon the visual function, experimental paradigms, and characteristics of the studied sample (see e.g., Jachim et al., 2015).

Mechanisms of the altered visual perception in ASD are poorly understood. One of multiple pathophysiological processes that may contribute to both sensory-perceptual and cognitive abnormalities observed in ASD individuals is the altered ratio between excitation and inhibition in neural networks (i.e., E/I ratio; Rubenstein and Merzenich, 2003; LeBlanc and Fagiolini, 2011; Yizhar et al., 2011). It has been recently proposed that the E/I imbalance affects perception and behavior in ASD through the impaired divisive normalization—a neural computational mechanism that balances the excitation of an individual neuron with inhibition reflecting the overall activity of the surrounding neural population (Rosenberg et al., 2015). This divisive normalization was implicated in a wide range of perceptual phenomena, including contrast saturation, and marginalization, a type of probabilistic inference that eliminates irrelevant information. Rosenberg and colleagues suggested that the abnormally low divisive normalization in individuals with ASD might result in a reduced influence of past experience (i.e., environmental statistics) on the processing of incoming sensory information.

Influence of past experience on neural activity of primary visual cortex is most spectacularly reflected in the phenomenon of the oblique effect, i.e., much better orientation discrimination along cardinal (vertical and horizontal) than along oblique axes, as reported in a neurotypical population (Appelle, 1972). The development of orientation selectivity depends on an interaction between innate neural mechanisms and visual experience (Tanaka et al., 2009). Within this framework the anisotropy of orientation discriminability (i.e., the “oblique effect”), which is suggested to be a consequence of the prevalence of vertical and horizontal orientations in the environment (Girshick et al., 2011), may reflect developmental plasticity of the visual cortex. Interestingly, the oblique effect is already evident in human infants within the first year of life (Held et al., 1979; Gwiazda et al., 1984; Jouen, 1985; Fang et al., 1997). After this early developmental period, the difference in acuity between oblique and cardinal axes was found to be relatively stable for at least 4 years (Birch et al., 1983) and is only weakly influenced by the later experience in adults (Vogels and Orban, 1985). At the neuronal level the oblique effect is explained by the higher proportion and sharper tuning of neurons responding to cardinal than to oblique orientations (Li et al., 2003).

According to the predictions of the deficient divisive normalization account, ASD individuals should have a reduced oblique effect due to atypically increased line orientation discrimination threshold along cardinal axes (Rosenberg et al., 2015). Given that the oblique effect is a basic feature of visual processing reflecting E/I balance, this effect's investigation in ASD subjects may shed light on the nature of visual sensory abnormalities frequently reported in this population. A predicted reduction of oblique effect in children with ASD, if found, could illuminate the abnormal experience-dependent plasticity as underlying causes of atypical developmental trajectories of perceptual skills observed in infants later diagnosed with ASD (Sacrey et al., 2014). To the best of our knowledge, no previous studies examined orientation discrimination abilities in children with ASD.

The current investigation's objective was to address this gap in the knowledgebase concerning ASD. The approach was to examine vertical and oblique line orientation discrimination sensitivity in children with ASD and in typically-developing (TD) control participants aged 7–15 years. The analysis was performed in a broad sample of verbal children with ASD who were able to understand and perform the task, as well as in TD and ASD groups closely matched on IQ. The reason for this approach was two-fold. On the one hand, the ASD sample that consists exclusively of participants with high IQ is non-representative with respect to general population of ASD individuals comprising about 70% of individuals with below average intelligence (IQ < 85) (Charman et al., 2011). On the other hand, inclusion of developmentally delayed participants raises the question to what extent the results of the psychophysical testing are affected by intellectual ability *per se*. Therefore, the examination of the carefully IQ-matched ASD and TD subsample allowed us to control for IQ effect, whereas the inclusion of ASD participants with mild intellectual disabilities—to prove the generalizability of the results. We expected that the line orientation thresholds along the cardinal axis will be enhanced in ASD, resulting in an abnormally reduced oblique effect irrespective of children's IQ.

## Materials and methods

### Participants

The initial sample included 40 boys with ASD and 38 typically developing (TD) boys recruited at rehabilitation centers affiliated with the Moscow University of Psychology and Education and from the local community, respectively. The exclusion criteria were a known chromosomal syndrome (e.g., Down Syndrome, Fragile X syndrome), or a diagnosed neuropsychiatric disorder other than ASD (e.g., epilepsy). The diagnosis of ASD was confirmed by an experienced psychiatrist and was based on the Diagnostic and Statistical Manual of Mental Disorder-5 criteria as well as an interview with the parents/caregivers. Additionally, parents of all children were asked to fill in the Russian translation of the Autism spectrum Quotient (AQ) for children (Auyeung et al., 2008). The TD children who had scores above the cut-off of 76 (*n* = 2) and children with ASD who scored below this cut-off (*n* = 2) were excluded from the analysis. All participants with ASD were verbal. Subject's IQ was assessed using the Kaufman Assessment Battery for Children KABC II (Kaufman and Kaufman, 2004). All children had normal or corrected to normal vision according to the available medical records. The analysis was repeated for a subsample of children carefully matched on IQ. Approximately half of our ASD subjects had IQ scores below 94, the lowest value in the TD sample. Therefore, as a first step of IQ-matching ASD subjects with IQ scores above 90 were selected. As the next step, each subject of this subsample (*n* = 12) was individually matched with a TD participant in a way that a pair's Kaufman composite scores could differ by no more than 5 points. If several TD subjects could be matched to a particular ASD participant, then the TD subject with the closest IQ and age values was taken.

The previous studies suggest significant sex differences in etiological factors and the behavioral manifestation of ASD (Lai et al., 2015). Since the gender ratio in ASD is highly biased toward boys (Baio, 2012), a recruiting of the sufficient number of ASD girls for testing of the effect of gender might be problematic. Therefore, in our study we focused only on male participants, leaving the question about generalizability of our results across gender for the future studies.

The investigation was approved by the local ethics committee of the Moscow University of Psychology and Education and was conducted in accordance with the ethical principles of the Declaration of Helsinki regarding human experimentation. All children provided their verbal consent to participate in the study and were informed about their right to withdraw from the study at any time during the testing. Written informed consent was also obtained from a parent/guardian of each child.

### Stimuli and procedure

**Figure 1:**
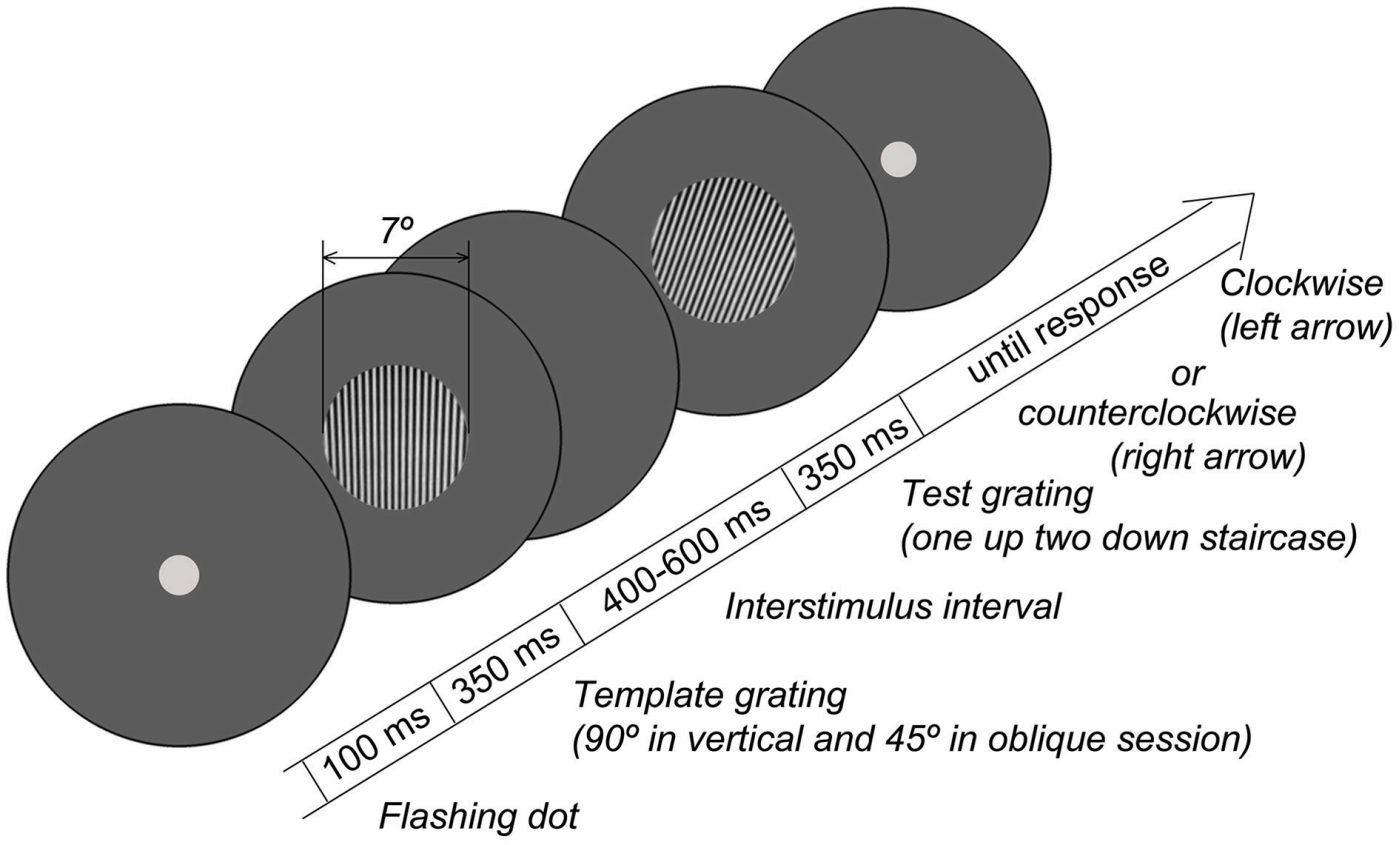
**Schematic diagram of the orientation discrimination task**.

The experimental procedure was similar to that previously used to measure orientation sensitivity in adults (Edden et al., 2009; Dickinson et al., 2014). Grating stimuli (circular; diameter 7°; spatial frequency 3 cycles/degree; 100% contrast) were created in MATLAB and presented using the PsychToolbox set of functions (Brainard, 1997) at a mean luminance of 3.3 Lux measured at eye-level. The stimuli were presented on 19″ W-LED Nec Multi Sync EA192M-BK monitor (resolution 1280 × 1024) controlled by the Mobile Intel® 945GM Express graphics chipset. Participants sat comfortably at a 60 cm distance from the monitor. The distance from the monitor, verticality of the head position, and adequacy of the task performance were all controlled by a researcher who sat next to the child. The room was dimmed, and a circular aperture (with diameter of 61 cm, and inner empty circle of 13 cm) was placed over the monitor to remove the external orientation cues, such as those from the edges of the screen. Orientation discrimination thresholds were measured using a two-alternative forced choice adaptive procedure. The experimental paradigm is schematized in Figure 1. Each trial started with the presentation of a central dot that flashed twice to attract the participant's attention. In 100 msec, two gratings were presented sequentially for 350 msec each, with an interval varied randomly between 400 and 600 msec. The orientation of the first (template) grating was 90° in the vertical condition and 45° in the oblique condition. The second grating was rotated either clockwise or counterclockwise relative to the template. The orientation difference between the gratings was adjusted using two interleaved one-up two-down staircases that converged on 71% correct performance either from clockwise or counterclockwise directions. Participants used a keyboard to report whether the second grating was rotated clockwise or counterclockwise relative to the first grating. The initial difference between the first and the second gratings was 15°. The initial step was 1°, which was reduced to 0.4° after the 2nd reversals and to 0.2° after the 4th reversals. The block continued until both staircases completed 7 reversals, typically lasting about 7 min. The thresholds were computed by averaging over the reversals, excluding the first two of each staircase. There were separate blocks for vertical and oblique conditions.

**Figure 2:**
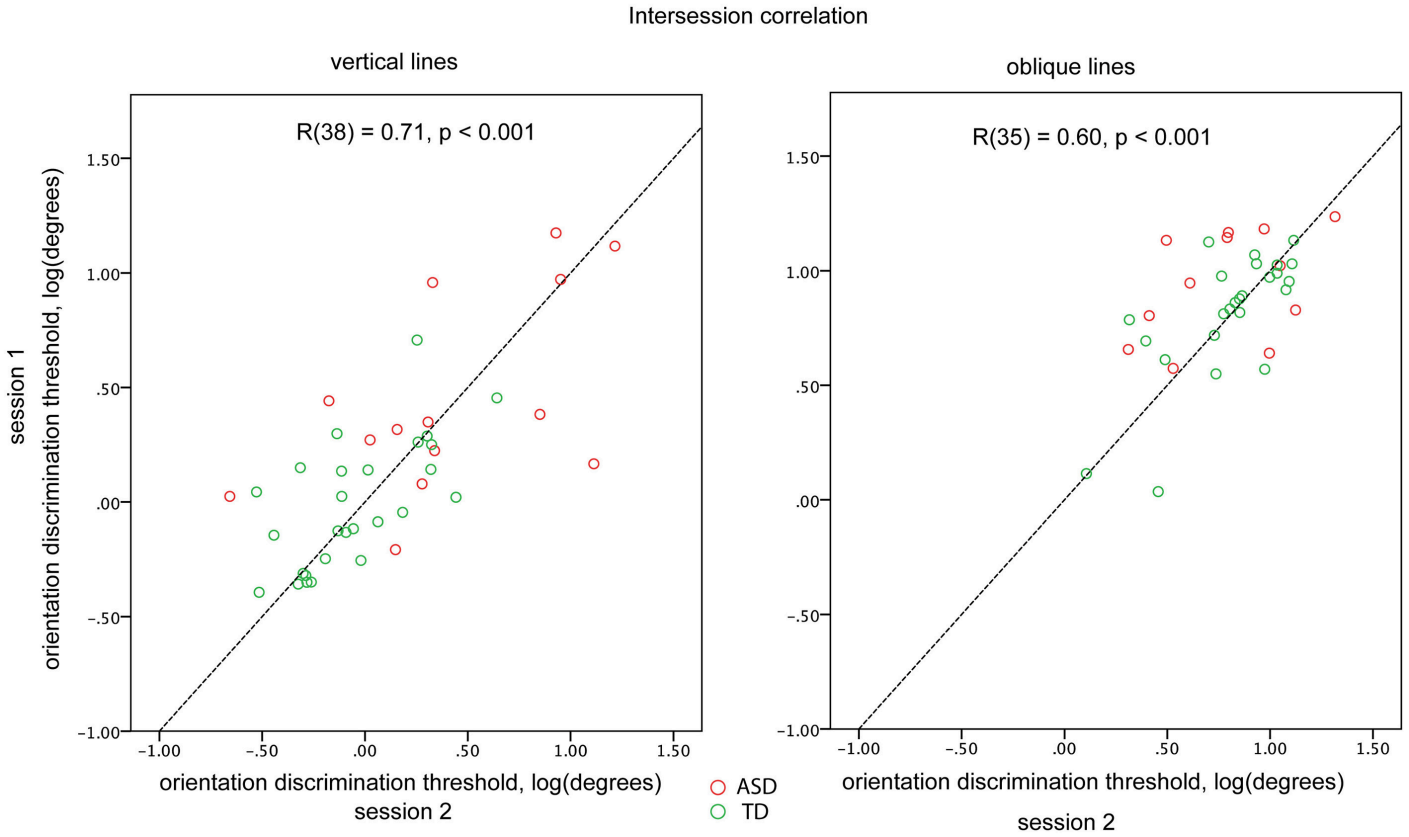
**Orientation discrimination thresholds obtained in different sessions for vertical (left plot) and oblique (right plot) conditions**. Individuals with ASD are represented by red circles and TD individuals by green circles. The dashed line corresponds to the *y* = *x* function.

For a subset of participants (14 ASD and 26 TD for the vertical and 12 ASD and 25 TD for the oblique condition) the blocks were administered twice to examine the reliability of the obtained thresholds. The first and the second administrations were separated by 10–30 min interval. There was a fairly high and highly significant inter-session correlation for both vertical [*R*_(38)_ = 0.71, *p* < 0.001] and oblique [*R*_(35)_ = 0.60, *p* < 0.001] conditions, when the ASD and TD participants were pooled together. Inter-session correlation coefficients did not differ significantly (*p* > 0.1) between vertical and oblique conditions. There were no significant differences in correlation coefficients between the TD and ASD participants (vertical: 0.58 and 0.65; oblique: 0.43 and 0.72, for ASD and TD, respectively; Figure 2). To ensure that the estimated reliability can be generalized to our full sample, we compared the obtained orientation discrimination thresholds—as well as IQ, AQ, and age between participants-who performed one vs. two experimental sessions. No significant differences in these parameters were found in either TD or ASD groups (all *p*s > 0.05).

The thresholds measured during the first and the second administrations did not differ for either the cardinal or oblique orientation conditions (both *p*s > 0.1), indicating the absence of effects of training and/or fatigue. Therefore, when available, the thresholds obtained in the two experimental sessions were averaged. The testing always started with the cardinal block that was followed by the oblique block. The order of the two remaining blocks was counterbalanced across participants.

The experimental procedure was explained to the participants using printed instructions depicting the stimuli. Before the experimental sessions, the participants performed a short training session that included only 10 trials and started with 20° initial orientation difference between the first and the second gratings, but otherwise was identical to the main experimental session. The experimenter monitored behavioral performance, providing help as necessary. If there were any concerns that the participant had not correctly understood the task, the explanation was repeated and the training session re-administered.

### Data analysis

**Figure 3:**
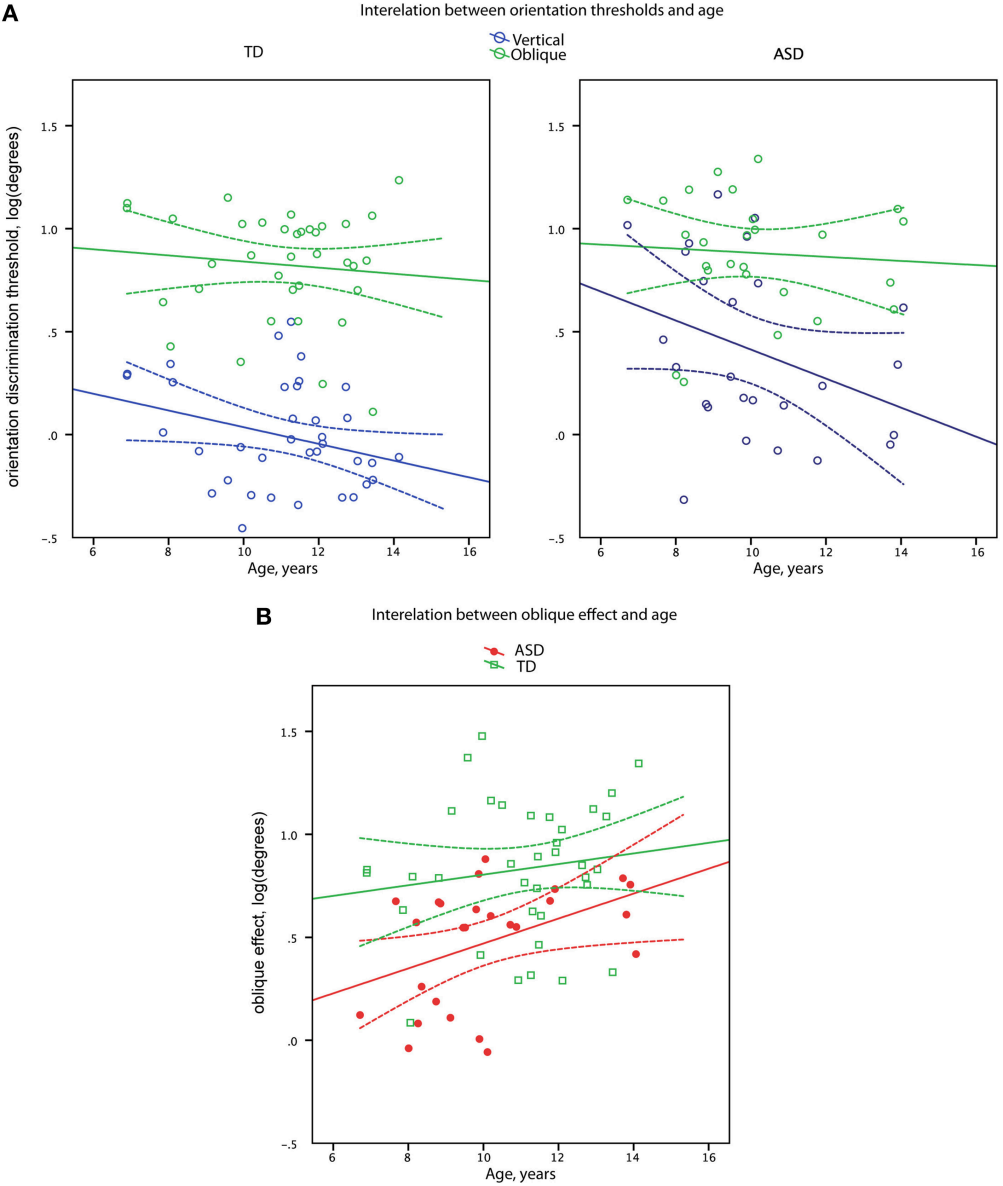
**Orientation discrimination thresholds (A) and oblique effect (B) as a function of age in boys with ASD and in (TD) boys**. Data points and regression lines are shown in blue for the oblique orientation and in green for the vertical orientation **(A)**. Data points and regression lines are shown in red for ASD and in green for TD groups **(B)**. Dashed lines denote 95% confidence intervals.

To normalize the data distribution we calculated the common logarithm of the orientation discrimination thresholds measured in degrees of visual angle. In neither the ASD nor the TD group (Shapiro-Wilk test, *p*s > 0.17) did the distribution of cardinal orientation discrimination thresholds deviate from normality. For oblique orientation the thresholds' distribution deviated from normality in the TD group (*p* = 0.02) yet not the ASD group (*p* = 0.39), as was mainly attributable to one TD outlier having a very low threshold of 1.7° (0.11 on the logarithmic scale; a value within 3 SD of the group mean 0.83 ± 3^*^0.26). Figures 3 and **5** confirm the absence of extremes among our participants. Parametric statistical analysis was thus appropriate.

An Analysis of Variance (ANOVA) with Orientation (Vertical vs. Oblique) manipulated as a within-participants factor and Group (ASD vs. TD) as a between-participants factor was used to examine main effects and their interaction. Planned *post-hoc* tests were used to follow the significant ANOVA effects. η^2^ was used to estimate the effect sizes. The oblique effect was calculated as the difference between log-transformed orientation discrimination thresholds for oblique and cardinal lines. Pearson product-moment coefficients were used to test for correlations between orientation discrimination thresholds and age, IQ, and AQ scores. Fisher's *Z*-test and Williams' *t*-test were used to compare correlation coefficients.

## Results

### Final sample

Twelve children with ASD were unable to complete the task and were excluded from the study. The final sample was thus comprised of 26 ASD and 36 TD boys. The ASD and TD groups did not differ in chronological age [*t*_(60)_ = 1.84, *p* = 0.07], but the mean IQ score was higher in the TD than in the ASD participants [*t*_(60)_ = 7.04, *p* < 0.001]. The analysis was repeated for the subsample of children closely matched on IQ. There was no significant age difference between the ASD and TD groups matched on IQ [*t*_(24)_ = 1.22, *p* = 0.24]. The information on participants included in the study is summarized in Table 1.

**Table 1:** **Demographic information for the IQ-matched and full samples: mean ± sd (range)**.

	**Age**	**IQ**	**Child AQ**
	**Full Sample**		
ASD (*N* = 26)	10.1 ± 2.0 (6.7–14.1)	91.8 ± 16.9 (68–122)	92.2 ± 12.1 (77–121)
TD (*N* = 36)	11.0 ± 1.9 (6.9–14.1)	117.7 ± 12.3 (94–141)	55.3 ± 13.1 (32–76)
	**IQ-Matched Sample**		
ASD (*N* = 12)	10.1 ± 2.1(7.6–13.8)	106.8 ± 10.3(92–122)	92.2 ± 11.5 (77–118)
TD (*N* = 12)	10.7 ± 1.9 (6.9–13.4)	106.0 ± 8.6 (94–119)	55.8 ± 15.2 (35–76)

### Effect of age

#### Orientation discrimination thresholds

Vertical line discrimination thresholds decreased with age in the combined sample [*R*_(60)_ = −0.37, *p* = 0.003] with the similar correlation coefficients in the TD and ASD participants (−0.27 and −0.34, respectively). In case of oblique line discrimination thresholds, no correlations with age were observed in either the combined sample [*R*_(60)_ = −0.10, *p* = 0.44] or separately in the TD and ASD participants (−0.09 and −0.07, respectively). Comparison of the age-related correlation coefficients for the oblique and cardinal orientations in the combined sample confirmed the presence of significant difference [*t*_(59)_ = 2.15, *p* = 0.03] between developmental trends for oblique and vertical line orientation discrimination skills.

#### Oblique effect

The magnitude of the oblique effect significantly increased with age in the full sample [*R*_(60)_ = 0.33, *p* = 0.009] with no significant difference (*Z* = 1.17, *p* = 0.24) between correlation coefficients in the TD and ASD participants (0.14 and 0.43, respectively), as depicted in Figure 3.

### Effect of IQ

#### Orientation discrimination thresholds

Vertical line discrimination thresholds did not correlate with IQ in either the TD [*R*_(34)_ = 0.10, *p* = 0.56], or the ASD sample [*R*_(24)_ = −0.20, *p* = 0.34]. Oblique line orientation threshold showed a moderate correlation with IQ in the ASD group [*R*_(24)_ = −0.40, *p* = 0.046], and there was similarly negative correlation for the TD group that was, however, a marginal trend [*R*_(34)_ = −0.31, *p* = 0.06].

#### Oblique effect

The oblique effect did not correlate with IQ in either of the groups [TD: *R*_(34)_ = −0.33, *p* = 0.053; ASD: *R*_(24)_ = −0.10, *p* = 0.63].

### Group differences

#### Full sample

**Figure 4:**
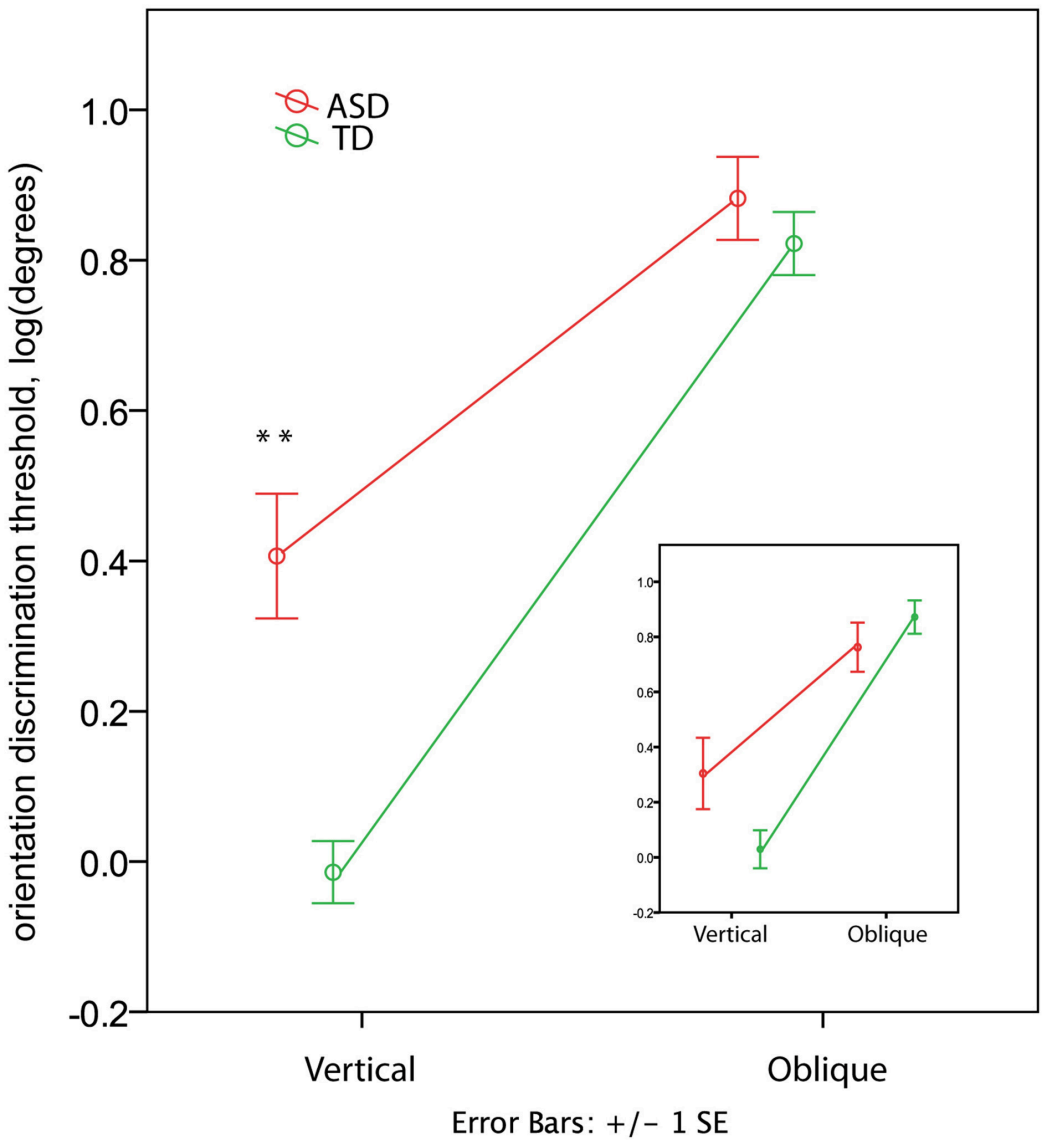
**Group differences in the oblique and vertical orientation discrimination thresholds (mean ± SE)**. The insert shows the thresholds for a subsample of 12 TD and 12 ASD participants matched by IQ. Note elevated vertical orientation discrimination threshold in children with ASD. ‘^**^’ corresponds to *p* < 0.001.

In the TD group the orientation discrimination thresholds measured in degrees were on average 1.4 (range 0.4–4.0) for the vertical axis and 8.3 (range 1.7–17.4) for the oblique axis. Although slightly higher, these values are comparable with those reported by Dickinson and colleagues for neurotypical adults using the same paradigm (see Figure 2 in Dickinson et al., 2014). In the ASD group the respective values were 4.4 (0.7–15.2) and 10.2 (3.1–23.7). The ANOVA analysis revealed a strong effect of Orientation [*F*_(1, 60)_ = 259.53, *p* < 0.00001, η^2^ = 0.812] that was due to the lower discrimination thresholds for vertical than for oblique orientation. The Group effect was also significant [*F*_(1, 60)_ = 11.80, *p* = 0.001, η^2^ = 0.164], indicating worse orientation discrimination ability in ASD than in TD children. This effect can be better explained by the Orientation by Group interaction [*F*_(1, 60)_ = 19.04, *p* = 0.001, η^2^ = 0.241], as illustrated in Figure 4. Although the oblique effect was highly significant in both the ASD [*F*_(1, 25)_ = 70.8, *p* < 0.00001, η^2^ = 0.739] and TD [*F*_(1, 35)_ = 224.1, *p* < 0.00001, η^2^ = 0.865] participants, this effect was substantially reduced in children with ASD as compared with TD due to their elevated vertical orientation discrimination threshold [*F*_(1, 60)_ = 22.30, *p* < 0.0001, η^2^ = 0.271]. The oblique line orientation discrimination threshold in ASD did not differ from normal [*F*_(1, 60)_ = 0.61, *p* = 0.43, η^2^ = 0.010].

#### IQ-matched sample

To eliminate the possibility that the group differences in the oblique effect were explained by differences in IQ between groups, we repeated the analysis for a smaller group of participants carefully matched on IQ (12 ASD and 12 TD, see Table 1). While the main Group effect became non-significant [*F*_(1, 11)_ = 0.036, *p* = 0.854, η^2^ = 0.003], the Orientation by Group interaction remained significant [*F*_(1, 11)_ = 8.858, *p* = 0.013, η^2^ = 0.446; see insert in Figure 4]. The direct between-group comparison of the oblique effect confirmed that the effect's magnitude was smaller in ASD than in TD boys [*t*-test for independent samples: *t*_(22)_ = 2.627, *p* = 0.015].

### Correlations between oblique and vertical orientation thresholds

**Figure 5:**
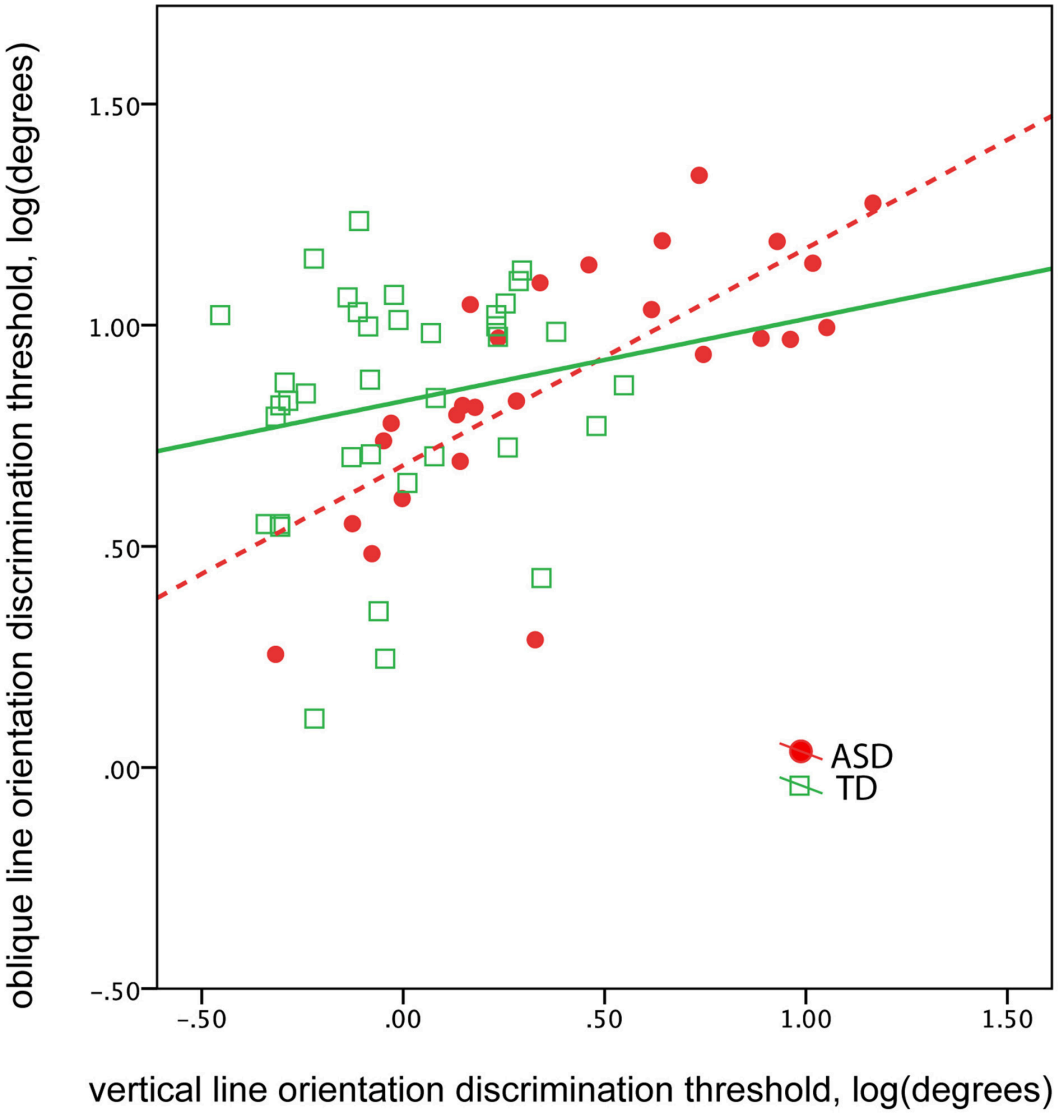
**The correlation between oblique and vertical orientation thresholds in TD boys (green) and in boys with ASD (red)**. Note greater correlation between the thresholds in the ASD group.

The abnormal vertical, yet not oblique, line orientation discrimination threshold found in children with ASD suggests a selective deficit in orientation sensitivity. This selective deficit may affect the correlation between vertical and oblique orientation discrimination thresholds. Indeed, the correlation between the thresholds obtained in vertical and oblique conditions was unexpectedly high in participants with ASD [ASD: *R*_(24)_ = 0.74, *p* < 0.0001; TD: *R*_(34)_ = 0.18, *p* = 0.29, Figure 5]. The difference between ASD and TD correlation coefficients was significant (*Z* = 2.83, *p* = 0.005). Controlling for IQ did not change correlations between the thresholds [partial correlation in ASD: *R*_(23)_ = 0.73, *p* < 0.0001; in TD: *R*_(33)_ = 0.23. *p* = 0.19]. These results suggest greater interdependence between orientation discrimination thresholds in ASD than in TD groups, regardless of intellectual ability.

### Orientation discrimination thresholds and autistic traits

A previous study reported a negative correlation between orientation discrimination thresholds relative to oblique axis and AQ scores in a large sample of neurotypical adults (Dickinson et al., 2014). Although we did not find differences in the oblique orientation discrimination threshold between children with and without ASD, we tested for a possible link between oblique orientation discrimination threshold and autistic traits separately in the TD and ASD children. No correlations between discrimination thresholds and AQ was found for either oblique [TD: *R*_(34)_ = 0.10, *p* = 0.54; ASD: *R*_(24)_ = 0.12, *p* = 0.57] or vertical [TD: *R*_(34)_ = −0.19, *p* = 0.25; ASD: *R*_(24)_ = 0.15, *p* = 0.46] line orientations. The oblique effect also did not correlate with the AQ [TD: *R*_(34)_ = 0.23, *p* = 0.17; ASD: *R*_(24)_ = −0.11, *p* = 0.60].

## Discussion

The novel contribution of this investigation was the first known assessment of line orientation discrimination thresholds in children with ASD. We found that regardless of IQ level, children with ASD had a reduced oblique effect—a difference in discrimination ability along vertical and oblique axes. The reduced oblique effect in children with ASD was explained by these children's impaired orientation discrimination ability along vertical yet not oblique axes.

### Potential confounds in measuring orientation sensitivity in ASD

Approximately half of our participants with ASD had mild intellectual disability. However, these participants' atypical performance cannot be fully accounted for by lower IQ, since the oblique effect was atypically reduced even in a group of high-functioning children with ASD and neither the magnitude of the oblique effect nor the vertical line orientation discrimination thresholds correlated with IQ scores in a broader ASD sample. Given that the reduction of the oblique effect in children with ASD was due to poor orientation sensitivity along cardinal but not oblique axes, the effect can hardly be explained by executive dysfunction or general inattentiveness, because such issues would non-specifically affect the psychophysical thresholds in the ASD population. However, the current investigation did not assess executive functions.

Another confounding factor that could influence our psychophysical results is the higher prevalence of ophthalmological problems in children with ASD comparing to TD peers (Simmons et al., 2009). The higher prevalence of strabismus, amblyopia, and anisometropia were reported among 44 patients with ASD aged 2–20 years (Black et al., 2013). These problems could lead to a range of symptoms including double or blurred vision that may potentially affect low-level visual functions including orientation sensitivity. Although our participants had no prominent ophthalmological problems according to the available medical records, the lack of thorough ophthalmological investigation is a limitation of the current investigation. On the other hand, many aspects of vision, including visual acuity, are unaffected in children with ASD. Such an assertion has stemmed from a recent comprehensive assessment of low-level visual functions (Milne et al., 2009), including visual acuity, stereoacuity, convergence, divergence, ocular motility, incidence of strabismus, and integrity of the optokinetic response. Another recent study (Kéïta et al., 2014) showed that contrast sensitivity for luminance-defined vertically-oriented sine-wave gratings was typical at low spatial frequencies (0.5, 1, 2, 4 cycles/degree) and even increased at 8 cycles/degree in high-functioning adolescents and adults with ASD as compared with control participants. These findings on generally unimpaired low-level visual functions in ASD do not refute a possibility that our results might be explained by some specific ophthalmological abnormality, which has not been yet tested in ASD studies. For example, individuals with ASD may have a selectively increased astigmatism along the vertical but not oblique axes. Although such a possibility does not seem very probable, we cannot rule-out this explanation completely. In view of the obtained results, thorough examination of refractive and corneal astigmatism along the primary meridians in ASD and TD subjects is definitely needed in the future research of the oblique effect. Since, until now, no studies reported increased rate of any ophthalmological problem in individuals with ASD, we can tentatively conclude that the observed selective deficit in orientation sensitivity most probably has a neurophysiological origin.

### Oblique effect and the theory of divisive normalization

Our finding of the reduced oblique effect in children with ASD is well in line with the predictions of the theory of abnormal divisive normalization (Rosenberg et al., 2015). The modeling study performed by Rosenberg and colleagues predicts that the reduced priors in the visual cortex of individuals with ASD would decrease the oblique effect: These priors would reduce the neural response to cardinal orientations and, to a lesser extent, increase the response to oblique orientations (Rosenberg et al., 2015, Supplementary Materials). While we did find the reduced sensitivity to vertical orientation in children with ASD, these children's sensitivity to oblique orientations appeared to be unchanged. It is possible that apart from the reduced priors, there is an additional factor that may non-selectively deteriorate orientation discrimination sensitivity to both cardinal and oblique lines in children with ASD. The link between this pathological factor and severity of ASD may explain the negative correlation between IQ and the oblique orientation discrimination threshold in our study. In the following sections we discuss the putative mechanisms affecting orientation discrimination sensitivity and reduction of the oblique effect in children with ASD.

### Orientation discrimination sensitivity in clinical studies

Although we are unaware of any previous studies assessing orientation discrimination ability in ASD population, a relation of the oblique effect to the presence of autistic traits has been studied in the neurotypical adults (Dickinson et al., 2014). The experimental procedure in the current investigation and in the study of Dickinson et al. was almost identical with few modifications: in our procedure the stimuli size was bigger (diameter 7 vs. 4° visual angle) and criteria for threshold convergence were milder (one up two down staircase vs. one up three down staircase in Dickinson et al., 2014). Noteworthy is that Dickinson and colleagues have found that the magnitude of the oblique effect negatively correlated with autistic traits due to an increased sensitivity to oblique line orientations in individuals with higher AQ scores, while no correlation between AQ and orientation sensitivity along vertical axis was reported. In other words, although the oblique effect in neurotypical adult participants with higher AQ scores was reduced similarly to ASD children in our study, the causes of this reduction were different. Contrary to the findings of Dickinson and colleagues, we did not find a correlation of AQ scores with either the oblique effect or the oblique orientation threshold: Such correlations were not found either for ASD or TD children. It was recently argued that the findings from neurotypical subjects cannot be directly extended to individuals with ASD diagnosis (Gregory and Plaisted-Grant, 2013). This might be the most plausible explanation for the discrepancy in findings in our study and in the study of Dickinson and colleagues. Other possible reasons for such a discrepancy might be the different age of the participants and/or slightly different experimental procedure. However, similarly to our study, another study of neurotypical adults failed to find a link between the amount of autistic traits and the participants' ability to discriminate orientations of oblique lines (Skewes et al., 2015).

We are not aware of any study examining orientation discrimination threshold in other neurodevelopmental disorders, therefore, the specificity of our finding for ASD population requires further examination. The available evidence from other clinical populations points to an impaired orientation sensitivity along an oblique yet not the cardinal axes. This impaired discrimination sensitivity to oblique line orientation was reported in clinical studies in patients with schizophrenia (Rokem et al., 2011) and migraine (Tibber et al., 2006). The authors explained the patients' impaired performance in the orientation discrimination task by a broader tuning of the orientation-selective cells due to diminished level of gamma-aminobutyric acid in visual cortex.

Indeed narrowing of visual receptive fields in mammals is attributed to maturation of GABA related inhibition (Li et al., 2012; Griffen and Maffei, 2014). In human subjects, Edden et al. (2009) reported that GABA levels in the primary visual cortex measured by magnetic resonance spectroscopy (MRS) correlated with the oblique but not with the vertical line orientation discrimination thresholds. In view of these findings, normal oblique but elevated vertical orientation discrimination thresholds in children with ASD cannot be explained by a global GABAergic deficit in their visual cortex. Arguably, consistent with this view a recent MRS study reported normal GABA to creatine (GABA+/Cr) ratio in the visual cortex in children with ASD (Gaetz et al., 2014). As we discuss below, the selective impairment of vertical orientation discrimination and a concomitant reduction of the oblique effect in children with ASD is likely to reflect a more specific deficit in neural mechanisms underlying preference for cardinal orientations.

### Developmental trajectories of orientation sensitivity and the oblique effect

The orientation discrimination thresholds for vertical but not oblique lines improved between 7 and 15 years of age (Figure 3). Since vertical orientations are widely represented in human visual environment, such selective improvement may reflect a prolonged environmental tuning of orientation sensitivity during development (Girshick et al., 2011). Importantly, the developmental improvement of vertical line orientation discrimination sensitivity was observed in both experimental groups, suggesting that the mechanisms involved in the environmental tuning of orientation discrimination sensitivity operating during childhood and adolescence are intact in individuals with ASD. On the other hand, although there was a developmental increase in oblique effect, the magnitude of the oblique effect was consistently less prominent in children with ASD than their TD peers (Figure 3), suggesting that the mechanisms underlying such reduction are likely to operate already very early during development.

Indeed, unlike orientation sensitivity—which has a protracted developmental course—orientation anisotropy seems to be mostly developed within the first year of life. Indeed, several studies using the preferential looking technique have reported that human infants have better visual acuity along cardinal than oblique axes already at 3–7 months (Jouen, 1985), 7 months (Held et al., 1979), or by age of 10 months (Gwiazda et al., 1984; Fang et al., 1997). After this early developmental period, the difference in acuity between oblique and cardinal axes was not found to change significantly throughout the period between 7 and 60 months with visual acuity being approximately 1/4 of an octave lower for oblique than for the cardinal gratings (Birch et al., 1983, but see Mayer, 1977 for different results on the anisotropy in contrast sensitivity). Moreover, this early formed anisotropy can hardly be substantially changed by the later experience. The extensive training in orientation discrimination leads to a decrease in discrimination threshold for oblique orientations until some stabilized level, which is still greater than that for the cardinal orientation (Vogels and Orban, 1985). On the other hand, a training to discriminate orientations along the vertical axis does not have a noticeable effect on the discrimination threshold (Vogels and Orban, 1985; Matthews and Welch, 1997). Moreover, the effect of perceptual learning does not transfer between orientations, suggesting that the learning-induced plastic changes are highly orientation-specific.

Given that the oblique effect in humans is a basic feature of visual processing with early developmental origin and a limited range of experiential modification, the reduction of the oblique effect in children with ASD cannot be explained by transitory developmental delay or limited visual experience.

Some insights into neural underpinning of the normal and aberrant development of cortical anisotropy comes from the animal studies that uncover mechanisms of the oblique effect in different species as well as at different developmental stages. In mice, the stripe-rearing (i.e., restriction of visual experience to contours of only one orientation) during the peak of critical period for orientation sensitivity leads to overrepresentation of the experienced orientation among neurons in the visual cortex. This overrepresentation is due to an instructive mechanism altering the orientation tuning of individual neurons (Kreile et al., 2011). A similar effect was observed in kittens (Sengpiel et al., 1999; Tanaka et al., 2009). Proving that during the critical period for orientation plasticity the neurons changed their preferred orientation to those overrepresented in the artificial visual input, the animal data directly demonstrate that environmental statistics contribute to the experience-dependent orientation plasticity. Further, the stripe-rearing findings suggest a presence of an early short-term critical period for orientation plasticity, similar to that found for ocular dominance, as well as of the residual orientation plasticity in adulthood. The mechanisms underlying the critical period plasticity and adult plasticity were suggested to be different (Tanaka et al., 2009; Yoshida et al., 2012). Indeed, the perceptual learning in adult monkeys sharpens orientation tuning of the relevant orientation-selective neurons but does not change the number of neurons tuned to a particular orientation (Schoups et al., 2001). Thus, the increase in the number of neurons tuned to cardinal orientations during the critical period may explain the early emergence, developmental constancy and life-long maintenance of the oblique effect. The above considerations suggest that the atypical visual anisotropy in children with ASD could emerge at a developmental stage overlapping with the critical period for orientation sensitivity, i.e., during the first months of life. Subsequent plastic changes in visual circuitry brought by experience-related learning, although might refine orientation tuning of the existing neurons across different orientations (Creutzfeldt and Heggelund, 1975; Tanaka et al., 2009; Yoshida et al., 2012), cannot fully compensate for the lack of superiority for cardinal orientation discrimination defined by mechanisms operating within the critical period.

We speculate that since the superior discrimination of cardinal orientations in typically developing children originates mainly from the neural events that are unique for the critical period of orientation sensitivity, the significant reduction of the oblique effect in children with ASD could be explained by a disrupted mechanism of the early experience-dependent learning that takes place during the critical period for orientation selectivity.

### A putative neural underpinnings of the reduced oblique effect in children with ASD

The development of orientation tuning at early ages is crucially dependent on inhibitory synaptic mechanisms and, more specifically, on maturation of parvalbumin (PV) positive inhibitory neurons (Lee et al., 2012). Our results are thus consistent with the E/I imbalance model of ASD (Rubenstein and Merzenich, 2003). The early experience-dependent plasticity relies upon functioning of N-methyl-D-aspartate (NMDA) receptors on PV-containing interneurons (Zhang et al., 2013). Importantly, a disruption of NMDA transmission during critical period precludes experience-dependent refinement of orientation specificity of visual neurons (Ramoa et al., 2001; Fagiolini et al., 2003; Tanaka et al., 2009). Fagiolini and colleagues suggested that development of orientation preference through sensory experience might depend on cascade of postsynaptic events specifically connected to NMDA receptors (Fagiolini et al., 2003). Since NMDA transmission was implicated in the of autism (Gandal et al., 2012; Lee et al., 2015), dysfunction of that neurotransmission during the critical period may reduce excitability of inhibitory neurons and decrease neural specialization for detection of statistically more probable orientations. The constitutive NMDA receptors dysfunction is particularly disruptive during infancy and is likely to affect predominantly cardinal orientations overrepresented in the environment, nonetheless, it may also influence sensitivity to other orientations. The abnormally high correlation between the oblique and vertical orientation discrimination thresholds in children with ASD in the current investigation (Figure 5) is in line with the presence of such a common pathological factor. The suggested effect of a constitutive disturbance of NMDA-related transmission on the basic visual functions in ASD individuals clearly merits further investigation.

Another factor that may lead to selective impairment of orientation discrimination along the vertical axis and to reduction of the oblique effect is disrupted intracortical connectivity. It has been argued that the visual orientation biases in the primary visual cortex cannot be accounted for solely by linear processes such as the feed-forward connections from lateral geniculate nucleus to the visual cortex and, accordingly, anisotropy must be driven by feedback from extrastriate visual areas (Li et al., 2003; Koelewijn et al., 2011). This hypothesis is supported by results of animal studies showing that a strength of the oblique effect in the cat's primary visual cortex is strongly modulated by excitatory feedback from the middle temporal visual area (Liang et al., 2007). Experiments incorporating recording of magnetic activity of the brain in humans suggest that the decreased feedback connectivity may be an important feature of autism (Khan et al., 2015; Kitzbichler et al., 2015). Therefore, feedback connectivity from the higher-order visual areas to the primary visual area (V1) may be another source of the reduced precision for vertical orientation in children with ASD.

### Implications for the theory of enhanced perceptual functioning in ASD

Mottron and colleagues have suggested that perceptual functioning may be enhanced in people with ASD due to the over-functioning of brain regions typically involved in primary perceptual functions (Mottron et al., 2006). This theory was partly based on results of Bertone et al. (2005) who observed the superior ability to differentiate between vertical and horizontal low-contrast luminance-defined gratings in individuals with ASD. Our finding of impaired orientation discrimination of high contrast vertical gratings in children with ASD contradicts this broadly formulated Enhanced Perceptual Functioning model and suggests that visual sensory abilities in individuals with ASD depend on the nature of the visual tasks. In particular, the discrimination of line orientation in the present study may rely on the different neurophysiological mechanisms than those involved in detection of low-contrast stimuli used by Bertone and colleagues. Indeed, blockade of GABAa inhibition in primary visual cortex does not affect contrast sensitivity of the neurons but dramatically reduces orientation and directional sensitivity (Katzner et al., 2011). On the other hand, cortical excitation through basal forebrain stimulation improves contrast sensitivity while decreases orientation selectivity (Bhattacharyya et al., 2013). Considering these physiological findings, the increased E/I ratio in individuals with autism (e.g., Rubenstein and Merzenich, 2003; LeBlanc and Fagiolini, 2011) would predict normal or higher than normal contrast sensitivity but an impaired discrimination of small difference in orientations of high-contrast, clearly seen lines. Thus, both the previous finding of enhanced contrast sensitivity (Bertone et al., 2005) and our present data on reduced vertical orientation sensitivity comply with the theoretical model of E/I imbalance in ASD. Our findings suggest that in visual cortex this putative imbalance has particularly adverse consequences for discrimination of cardinal (vertical) orientations.

To sum up, our study revealed that irrespective of intellectual level the oblique effect was significantly reduced in boys with ASD as compared to the typically developing boys. This reduction was due to the poor ability of children with ASD to discriminate orientations along the vertical axis, whilst their ability to discriminate oblique orientations was normal. The selective impairment of discrimination along the vertical axis seems to be present from an early age and may result from abnormal experience-dependent plasticity during the sensitive period for orientation tuning and/or from abnormal connectivity of the primary visual cortex. The observed impairment of orientation discrimination ability may provide useful biomarkers pointing to a particular ASD-related neural deficit. Further, given the early developmental origin of the bias in typical orientation sensitivity, the reduction of that bias in the ASD population suggests that the early signs of ASD might include behaviors that fall outside the range currently used in prospective longitudinal studies of infants ‘at risk’ for autism (Landa et al., 2013). While the available findings in the high-risk infants who developed autism point to generally intact psychomotor development at 6 months of age, the first manifestations of abnormal perceptual learning in visual domain may occur prior to the appearance of overtly abnormal behavioral patterns. Further investigations are needed to test this prediction. Our results not only contribute to a better understanding of the mechanisms underlying ASD, but also provide important information about the normal and pathological development of the basic visual functions in humans.

## Author contributions

TS, EO, and OS substantially contributed to the conception and design of the work, as well as to analysis and interpretation of the data; IG, MD, and OS substantially contributed to the data acquisition and analysis. All authors participated in drafting the work or revising it critically for important intellectual content, approved the final version for publication, and provided an agreement to be accountable for all aspects of the work in ensuring that questions related to the accuracy or integrity of any part of the work are appropriately investigated and resolved.

## Funding

The study has been supported by Russian Science Foundation grant #14-35-00060 and the charity foundation for autism “Way out.”

### Conflict of interest statement

The authors declare that the research was conducted in the absence of any commercial or financial relationships that could be construed as a potential conflict of interest.

## Supplementary material

The Supplementary Material for this article includes the resulting data matrix and can be found online at: http://journal.frontiersin.org/article/10.3389/fnins.2015.00512
